# Supplementary material for: Hurricane-induced demographic changes in a non-human primate population
Source: R Soc Open Sci. 2020 Aug 19;7(8):200173. doi: 10.1098/rsos.200173 (PMC7481679; doi:10.1098/rsos.200173)
Supplement: Supporting Information [file rsos200173supp1.docx]

**Supplemental Material**

To determine whether our annual matrix models based on stable stage theories accurately represent the observed changes in stage distributions, we used the observed structure of females and the annual matrices **Q***_t_* from 1973 to 2018 to compare the observed population structure with the projected structured one year after a major hurricane event. We also computed the stable stage distribution vector *w* and compare it with the observed and projected population structures.

**Figure S1**. Observed and projected stage-specific proportion of Cayo Santiago female rhesus macaques following major hurricanes. The observed structure represents the actual population structure during the hurricane year. The stable stage distribution (SSD) represents the projected SSD from eigen analysis following a hurricane year. The observed projection represents the projected population following a hurricane year. I = infants; Y = yearling; J = juvenile; NB = nonbreeders; FB = failed breeders; B = successful breeders.

**TABLE SI.** Life table response experiment (LTRE) showing the matrix of contributions **C** of each stage class transition of Cayo Santiago rhesus macaque females to changes in population growth rate following hurricane years.

|  |  | Reproductive stages | | | | | |
| --- | --- | --- | --- | --- | --- | --- | --- |
|  | I | Y | J | NB | FB | B |  |
| I | 0 | 0 | 0 | 0 | 0 | 0 |  |
| Y | 0 | 0 | 0 | 0 | 0 | 0 |  |
| J | 0 | 0.0002 | 0 | 0 | 0 | 0 |  |
| NB | 0 | 0 | -0.0004 | 0.0007 | 0.0034 | 0.0050 |  |
| FB | 0 | 0 | -0.0003 | 0.0060 | -0.0005 | 0.0014 |  |
| B | 0 | 0 | -0.0011 | **-0.0135** | -0.0023 | -0.0111 |  |

**Note**: *I* = infant; *Y* = yearling; *J* = juvenile; *NB =* nonbreeder*, FB =* failed breeder*, B =* successful breeder. Bold number represents the transition with the highest relative contribution to the population growth rate.

**Table SII**: Model selection for generalized additive mixed models testing for variability in age-specific fertility of Cayo Santiago rhesus macaque females as a function of treatment with population density and individual ID as random effects.

| Model ~ | df | logLik | AICc | delta | weight |
| --- | --- | --- | --- | --- | --- |
| treatment + s(age) | 6 | -6513.8 | 13039.5 | 0.00 | 0.70 |
| s(age) | 5 | -6515.6 | 13041.3 | 1.72 | 0.30 |
| treatment + s(age by treatment) | 8 | -6527.5 | 13070.9 | 31.4 | 0.00 |
| s(age by treatment) | 7 | -6529.3 | 13072.6 | 33.1 | 0.00 |
| null (intercept-only model) | 3 | -7415.8 | 14837.6 | 1798 | 0.00 |

**Note**: treatment = hurricane year, non-hurricane year; s(age) is the smooth term, s(age by treatment) is the interaction in the smooth term.

**Table SIII**. Model coefficients of the top model describing the variability in age-specific fertility of Cayo Santiago rhesus macaque females as a function of treatment with population density and individual ID as random effects.

| **Factor** | **Estimate** | **SE** | **z value** | **P value** |
| --- | --- | --- | --- | --- |
| *Parametric coefficients* |  |  |  |  |
| intercept | 0.802 | 0.180 | 4.465 | <0.0001 |
| treatment non-hurricane | 0.368 | 0.186 | 1.976 | 0.0481 |
| *Smooth parameter* |  | **EDF** | **Chi.sq** | **P value** |
| age |  | 8.437 | 1473 | <0.0001 |

**Figure S2**: Partial effects of predictors of top model.
